# Salivary cortisol and α-amylase: subclinical indicators of stress as cardiometabolic risk

**DOI:** 10.1590/1414-431X20165577

**Published:** 2017-02-06

**Authors:** S. Cozma, L.C. Dima-Cozma, C.M. Ghiciuc, V. Pasquali, A. Saponaro, F.R. Patacchioli

**Affiliations:** 1 Department of Otorhinolaryngology, School of Medicine, University of Medicine and Pharmacy “Grigore T. Popa”, Iasi, Romania; 2 Department of Internal Medicine, School of Medicine, University of Medicine and Pharmacy “Grigore T. Popa”, Iasi, Romania; 3 Department of Pharmacology, School of Medicine, University of Medicine and Pharmacy “Grigore T. Popa”, Iasi, Romania; 4 Department of Psychology, Sapienza University of Rome, Rome, Italy; 5 Department of Physiology and Pharmacology “V. Erspamer”, Sapienza University of Rome, Rome, Italy

**Keywords:** Salivary stress biomarkers, Cardiometabolic risk (factors), Stress, Hypothalamic-pituitary-adrenal axis, Sympathetic adrenomedullary system

## Abstract

Currently, the potential for cardiovascular (CV) stress-induced risk is primarily based on the theoretical (obvious) side effects of stress on the CV system. Salivary cortisol and α-amylase, produced respectively by the hypothalamus-pituitary-adrenal (HPA) axis and the sympathetic-adrenomedullary (SAM) system during stress response, are still not included in the routine evaluation of CV risk and require additional and definitive validation. Therefore, this article overviews studies published between 2010 and 2015, in which salivary cortisol and α-amylase were measured as stress biomarkers to examine their associations with CV/CMR (cardiometabolic risk) clinical and subclinical indicators. A comprehensive search of PubMed, Web of Science and Scopus electronic databases was performed, and 54 key articles related to the use of salivary cortisol and α-amylase as subclinical indicators of stress and CV/CMR factors, including studies that emphasized methodological biases that could influence the accuracy of study outcomes, were ultimately identified. Overall, the biological impact of stress measured by salivary cortisol and α-amylase was associated with CV/CMR factors. Results supported the use of salivary cortisol and α-amylase as potential diagnostic tools for detecting stress-induced cardiac diseases and especially to describe the mechanisms by which stress potentially contributes to the pathogenesis and outcomes of CV diseases.

## Introduction

Cardiometabolic risk (CMR) refers to risk factors that increase the likelihood of experiencing vascular events or of developing metabolic disease (1,2). In addition to traditional cardiovascular (CV) risk factors (age, gender, family history, hypertension, dysglycemia, dyslipidemia, and smoking), CMR factors include abdominal obesity, insulin resistance, inflammation, lack of consumption of fruits and vegetables, sedentary lifestyle, and especially stress, an important component of modern life that has become a significant health problem in the general population (3,4).

Currently, we use the word “stress” to describe the feeling of being overwhelmed by the psychophysical stress-induced challenges of daily life; this feeling may be highly adaptive from an evolutionary point of view because it allows us to cope with similar circumstances in the future. For the sake of brevity and to avoid delving too far into the details of the evolution of the concept of stress that has occurred over the past few centuries, we will refer to the modern and very comprehensive proposal that was put forth by Bruce McEwen to explain the complexity of the stress response (5–8 [Bibr B09] [Bibr B10]). This proposal can be synthesized by the expressions “to be stressed” and “to be stressed out”, which distinguish good stress challenges from bad stress challenges. According to McEwen’s proposal, the pathophysiology of the stress response can be described by the concept of allostasis, which is the process of achieving stability (or homeostasis) through physiological or behavioral change. Allodynamic processes can be adaptive in the short term (allostasis) and maladaptive in the long term (allostatic load). The identity of the factors that determine the threshold between adaptive and maladaptive responses to stressors remains an open question for researchers in the field of stress. This question highlights the need to search for predictive biomarkers of the risk of developing stress-related diseases.

Activation of the hypothalamic-pituitary-adrenal (HPA) axis and sympathetic adrenomedullary (SAM) system is essentially an adaptive mechanism that enables the human body to maintain physiological stability in response to general stress signals. Complex reciprocal counterbalances between the HPA axis and SAM system have been described, and stress-induced chronic stimulation and dysregulation of these systems may cause metabolic abnormalities (8–13).

Substantial evidence indicates that chronic elevation of cortisol levels and dysfunction of the feedback system within the HPA axis play a prominent role in stress responses (14,15). However, the opposite has also been clearly shown as the hyporesponsive HPA axis has been linked to increased susceptibility to chronic illness (16–18 [Bibr B19]).

Salivary levels of free cortisol are characterized by circadian fluctuation; concentrations in the morning are significantly higher than those in the evening. The cortisol awakening response (CAR) refers to the typical production of cortisol that occurs upon awakening. As part of a cycle within a cycle, the CAR reflects the changes in cortisol concentration that occur during the first hour after waking from sleep in the morning (11 [Bibr B12],^18^–21).

Activation of the SAM system may induce pathophysiological changes in cardiovascular activity that range from a mere increase in heart rate (HR), blood pressure (BP) and free fatty acids in healthy subjects to the induction of angina, myocardial infarction, ventricular arrhythmia and acute heart failure in patients with significant coronary lesions (22,23). The search for a “cortisol-like” non-invasive and easily obtainable marker of SAM activation led to the identification of salivary α-amylase as a promising candidate because its secretion is under strong neurohormonal control (24–26).

Currently, the potential for CV stress-induced risk is primarily based on the theoretical (obvious) side effects of stress on the cardiovascular system. Furthermore, while CV/CMR factors are routinely assessed in clinical practice, saliva-based biomarkers produced by HPA axis/SAM system (dys)function during stress response are still not included in the routine evaluation of cardiovascular risk and require additional and definitive validation. Therefore, this article overviews studies published between 2010 and 2015 related to the use of salivary cortisol and α-amylase as subclinical indicators of stress, and as CV/CMR factors, including studies that emphasized methodological biases that could influence the accuracy of study outcomes.

## Material and Methods

**Figure 1:**
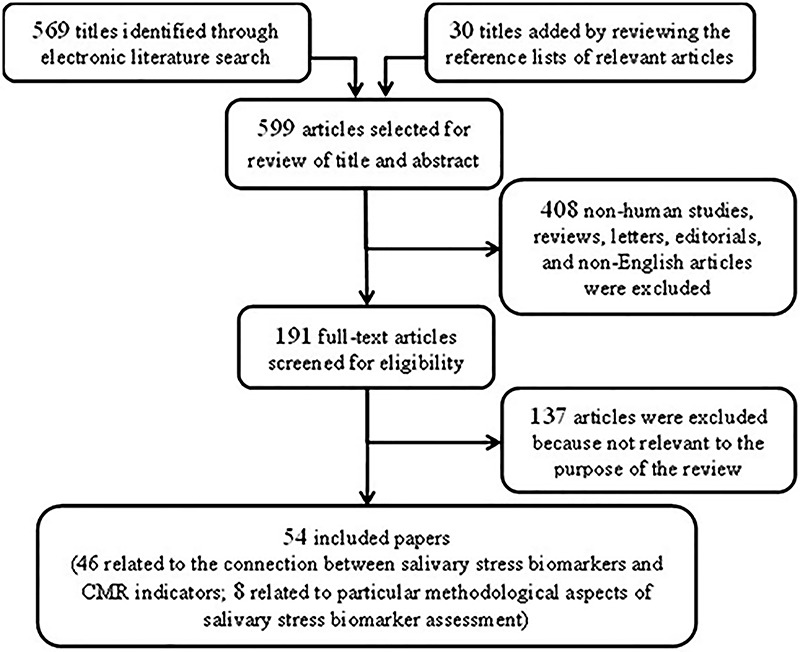
Literature selection method.

Eligible studies were original research articles published in peer-reviewed journals between January 2010 and December 2015 and identified through searches of the PubMed, Web of Science and Scopus electronic databases (27). We included studies if they involved human subjects in which salivary cortisol and α-amylase were measured as stress biomarkers to examine the associations with CV/CMR clinical and subclinical indicators. The term “cardiometabolic risk (factor)” was paired with “stress”, “psychological stress”, “stress hormones”, “salivary cortisol”, “salivary α-amylase”, “hypothalamus-pituitary-adrenal (HPA) axis ”, and “sympathetic adrenomedullary (SAM) system”. A flow chart describing the process of study identification is shown in Figure 1. During Skype conferences, all coauthors did the search through repeated use of the words in different combinations. Studies were included only if they assessed stress-induced response either to laboratory challenges, psychosocial items and/or diseases in different populations by measuring salivary cortisol and/or salivary α-amylase as well as clinical/subclinical indicators of CV/CMR.

The initial search yielded 569 titles. In addition, 30 supplementary titles were included by scanning the reference lists of retrieved papers. All abstracts were independently read by each coauthor: 408 non-human studies, reviews, letters, editorials, and non-English language reports were excluded. From the remaining 191 abstracts, all full manuscripts were gathered and were independently reviewed by each coauthor for key information. One hundred and thirty-seven studies were excluded because they were not relevant to the purpose of the review, since: a) they did not report salivary cortisol and/or α-amylase assessment, or b) because they evaluated stress response through psychometric tools only, or c) they considered the occurrence of stressful events itself as a CV/CMR factor without any salivary stress biomarker assessment.

All coauthors independently assessed eligibility by reviewing full text articles; if any coauthor was unsure whether an article met eligibility criteria, the article was discussed among the research team and full agreement was always reached. As shown in the flow chart of the literature selection process (Figure 1), 54 key articles were ultimately identified to be reviewed in this paper. Among these key articles, 46 were related to the stress-induced modification of the HPA axis and SAM system associated with variation in subclinical and clinical indicators of CV/CMR, and 8 addressed crucial methodological issues.

## Results

Supplementary Table S1 includes stressor, CV/CMR subclinical and clinical indicators, salivary biomarker measured, and a summary of the outcomes for each selected study. Sources of stress varied across studies. Stressors included mental task challenges, job strain and work shift, sport competition, effort test, ambulatory surgical stress, hypobaric hypoxia, and hypoxia and sleep fragmentation induced by obstructive sleep apnea in obese subjects.

Researchers studied salivary cortisol (11,28,30–34,37–40 [Bibr B41],^42^–47,49–54,56–68,70,71) and/or salivary α-amylase response to stress (11,29,32,33,35,36,40–42 [Bibr B43],^44^ [Bibr B45] [Bibr B46],^48^,^49^,^52^,^53^,^55^,^59^ [Bibr B60] [Bibr B61] [Bibr B62] [Bibr B63],^61^,^63^,^66^,^69^–^72^) for their association with CV/CM items. A substantial number of these studies showed significant correlations between salivary cortisol and/or salivary α-amylase and clinical/subclinical CV/CMR indicators (28,29,31,32,36,37,44,50,55). Several studies have demonstrated the association between stress-induced salivary cortisol and/or salivary α-amylase modifications and CV/CMR clinical/subclinical indicators (11,35,47,70,71).

Numerous methodological factors (biological and procedural/analytical) can influence saliva-based human neuro-endocrine measurements and, consequently, can dramatically compromise the accuracy and validity of research.

Table 1 lists studies providing an overview of the methodological recommendations that must be considered to prevent the danger of obtaining biased results when assessing salivary cortisol and/or salivary α-amylase as stress biomarkers. For example, nonadherence to the saliva collection protocol or invisible traces of blood can interfere with the results of saliva testing (78). Ecological momentary assessment studies provide reproducible and reliable evaluation of stress biomarkers that can be even improved through multiple sampling (20,73,76,78) and that, in contrast to blood sampling, can be easily achieved through saliva-based testing strategy. However, food intake should be restricted to 1–2 h prior to saliva sampling. Water intake does not affect test results, although coffee/alcohol and other drinks are not recommended (78). In addition, all other factors that influence salivary flow rate must be taken into account especially when salivary α-amylase is assayed as a non-invasive marker for SAM system activity (25). Other efforts to reduce bias include the development of specific and standardized analytical tools (74), the establishment of defined reference intervals, and the implementation of round-robin trials (75–77). However, the lack of compliance that sometimes occurs with outpatient saliva donors requires strict standardization of both collection and analysis methods to improve the possibility of comparing salivary biomarker data (20,73,76,78).

**Table:**
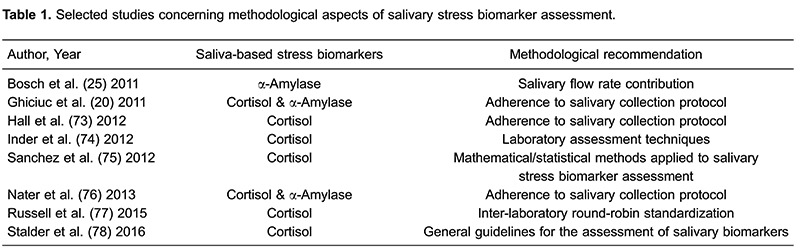

## Discussion

The purpose of this study was to provide a literature overview regarding the use of saliva-based stress biomarkers to explore the association of stress with CV/CMR indicators. Physiological stress response also includes a general arousal component that is associated with reactivity of the HPA axis/SAM system and is respectively measurable via salivary cortisol and via autonomic nervous system indices such as salivary α-amylase.

Articles have reported and discussed individual salivary cortisol and/or α-amylase differences in response to acute stress challenge (laboratory stress, i.e., under controlled conditions) that may be useful to underlie some of the subjective differences in CV/CM stress-induced vulnerabilities in healthy populations as well as in people with advanced CV/CM diseases (28,50,64). We have also reported studies supporting the connection between stress-induced allostatic (over)load and the appearance of more or less severe CV/CM disorders (37,47,50,51,53,56,57,64,65).

We found that the biological impact of stress measured by salivary cortisol and α-amylase was associated with CV/CMR factors. Taken together, the studies included in the present literature review suggest that changes in salivary cortisol and α-amylase concentrations, as well as their diurnal fluctuations, may have general implications for providing further biological mechanisms by which stress (overload) can be considered itself as a CV/CMR factor.

In agreement with previous speculations that HPA axis/SAM system dysfunction can potentially result in CV/CM diseases (79–82), the reports included in this review showed that several patterns of (acute/chronic) stress-induced production of salivary cortisol and/or salivary α-amylase are associated with abnormal HPA axis and SAM system activities, often concomitant with an unhealthy lifestyle, and potentially having detrimental effects on the CV system.

Monitoring activities of the SAM system and HPA axis is particularly important for the study of adaptive mechanisms of the autonomic and HPA stress resilience (32).

Salivary free cortisol is a well-known marker of HPA axis activity and plays a crucial role in an organism’s efforts to respond/adapt to stressors (15). The major advantage of salivary α-amylase measurements over other parameters that reflect SAM system activity (i.e., CV activity measures or skin conductance) is that it is measured in saliva and therefore allows ecological momentary assessments.

Although saliva has not yet become a common sample source for the analysis of saliva-based stress biomarkers, this evolving field of research represents a reliable method of investigating HPA axis and SAM activity, providing a means to avoid the stressful event of venipuncture and offering the possibility of self-collection by subjects.

Previous studies from other groups as well as from our own have shown that the collection of saliva provides a noninvasive, stress-free, reliable source for monitoring the human body's stress response, even in real life context, in different pathophysiological conditions without requiring the assistance of medical staff. Since 1983 (84) and thereafter, salivary cortisol concentration was found to be directly proportional to the serum unbound cortisol concentration both in normal men and women and in women with elevated cortisol-binding globulin. The correlation was excellent in dynamic tests of adrenal function (dexamethasone suppression, ACTH stimulation), in healthy subjects and in patients with adrenal insufficiency, in tests of circadian variation and in randomly collected samples. Women in the third trimester of normal pregnancy exhibited elevated salivary cortisol throughout the day. The rate of equilibrium of cortisol between blood and saliva was very fast, being less than 5 minutes. Since only free levels of cortisol are detected in saliva, salivary cortisol is suggested to be a more appropriate measure for the clinical assessment of adrenocortical function than serum cortisol (11,18,20,25,26,37,67,70,74,76,83–85).

Regarding salivary α-amylase, this biomarker is measured exclusively in saliva. During recent years, a growing interest emerged in using salivary α-amylase as a non-invasive, surrogate marker for sympathetic activity. Salivary α-amylase has been proposed as a sensitive biomarker for stress-related changes in the body that reflect the activity of the sympathetic nervous system, and a growing body of research is accumulating to support the validity and reliability of this parameter. Numerous studies applying stress protocols have demonstrated that salivary α-amylase is highly sensitive to stress-related changes. This field of research is still in its early stages. However, the studies included in our review further support the evidence that the employment of salivary α-amylase as an indicator of the SAM system (dys)-regulation is promising. Nevertheless, considerable long-term effort is still required for this approach to receive acceptance, especially by clinicians. Thus, actions to improve reliability can assist researchers in reducing measurement outcome variance of saliva-based stress biomarkers assessment and increase the validity of provided pathophysiological data, contributing to improving the interpretation and understanding of study results.

The main message of this overview is in support of the use of saliva-based stress biomarker assessment, not only in research but also in clinical practice. Indeed, in our literature review, we did not identify a single clear objection to the use of saliva as a diagnostic fluid, although we did identify strict methodological recommendations to avoid factors that influence and add variance to saliva-based stress biomarker measurement outcomes.

Stress-induced symptoms are malleable; they can affect different body systems and overlap between one type of disorder and another. Therefore, stress management strategies are frequently complex and need to be matched to the requirements of individual patients, beginning with the presumably positive diagnosis of a stress-induced disorder and continuing through rehabilitation and training to cope with distress. The use of validated saliva-based biomarkers as indicators of stress-induced body system vulnerability (allostatic overload) could support patients in improving their strategies to cope with challenging life events, through non-pharmacological approaches (11,58,86–88).

## Supplementary Material

Click here to view [http://bjournal.com.br/supplementary_material/5577.pdf].
